# Potential adverse effects on animal health and performance caused by the addition of mineral adsorbents to feeds to reduce mycotoxin exposure

**DOI:** 10.1007/s12550-019-00375-7

**Published:** 2019-09-13

**Authors:** Christopher T. Elliott, Lisa Connolly, Oluwatobi Kolawole

**Affiliations:** Institute for Global Food Security, School of Biological Sciences, Queens University Belfast, Belfast, UK

**Keywords:** Mycotoxins, Mineral adsorbents, Toxicity, Health effects, Feed safety

## Abstract

The contamination of feed with mycotoxins is a continuing feed quality and safety issue, leading to significant losses in livestock production and potential human health risks. Consequently, various methods have been developed to reduce the occurrence of mycotoxins in feed; however, feed supplementation with clay minerals or mineral adsorbents is the most prominent approach widely practiced by farmers and the feed industry. Due to a negatively charged and high surface area, pore volume, swelling ability, and high cation exchange capacity, mineral adsorbents including bentonite, zeolite, montmorillonite, and hydrated sodium calcium aluminosilicate can bind or adsorb mycotoxins to their interlayer spaces, external surface, and edges. Several studies have shown these substances to be partly or fully effective in counteracting toxic effects of mycotoxins in farm animals fed contaminated diets and thus are extensively used in livestock production to reduce the risk of mycotoxin exposure. Nevertheless, a considerable number of studies have indicated that these agents may also cause undesirable effects in farm animals. The current work aims to review published reports regarding adverse effects that may arise in farm animals (with a focus on pig and poultry) and potential interaction with veterinary substances and nutrients in feeds, when mineral adsorbents are utilized as a technological feed additive. Furthermore, results of in vitro toxicity studies of both natural and modified mineral adsorbents on different cell lines are reported. Supplementation of mycotoxin-contaminated feed with mineral adsorbents must be carefully considered by farmers and feed industry.

## Introduction

Feed is an integral part of the food chain, and it plays an important role in the growth, welfare, and productivity of farm animal as well as in the composition, safety, and quality of livestock products (milk, meat, and eggs) in the food supply chain (Guerre 2016; Makkar 2016). A complete or finished feed is manufactured from a mixture of raw materials of plant, animal, industrial, and pharmaceutical origin, and formulated to achieve a range of objectives in animals regarding health and performance (Ittiphalin et al. 2015; Guerre 2016). A recent global feed survey shows world compound feed production has reached 1.1 billion metric tons; China, USA, Brazil, Russia, India, Mexico, Spain, and Turkey represent the top eight countries responsible for 55% of total global feed production (Alltech 2019). Furthermore, commercial production of animal feeds takes place in 144 countries and generates an annual turnover of more than US$400 billion (IFIF 2018). This is expected to increase, as the industry is under pressure to increase the amount of safe and nutritious feed, to meet the global demand for livestock products (Makkar and Ankers 2014). However, one of the greatest challenges facing farmers and the feed industry is the occurrence of mycotoxins in feed ingredients (Li et al. 2014; Pinotti et al. 2016). Mycotoxins in feed are a potential risk for animal performance, health, and the safety of foods of animal origin. More than 400 mycotoxins have been identified; however, only few have attracted scientific and regulatory interest due to the huge difficulties of collecting sufficient data in terms of frequency of occurrence and toxicity. The regulated mycotoxins in many parts of the world include aflatoxin B1 (AFB_1_), deoxynivalenol (DON), ochratoxin A (OTA), zearalenone (ZEN), fumonisin B1 (FB_1_), and trichothecenes A– (T-2 and HT-2) (Zain 2011). These mycotoxins are produced by *Aspergillus*, *Fusarium*, and *Penicillium* species (Sweeney 1998) and their production is influenced by factors such as agronomic practice, pre- and post-harvest climate conditions (temperature, moisture level and carbon dioxide) (Pitt and Miller 2016; Gilbert et al. 2017). Hence, contamination can occur during crop growth, storage, and transportation (Mannaa and Kim 2017).

A number of mycotoxins surveys have been carried out to investigate the worldwide occurrence of mycotoxins in feed materials such as wheat, maize, soybean meal, and dried distillers’ grains (Monbaliu et al. 2010; Rodrigues and Naehrer 2012; Streit et al. 2013; Kosicki et al. 2016). The toxic effects of mycotoxin on animal health is termed mycotoxicosis, the degree of toxicity depends on the type of mycotoxins, level ingested, exposure time, breed, age, sex, health status of animal, and other stress factors (Zaki 2012; Khatoon 2016; Ostry et al. 2016; Dellafiora and Dall’Asta 2017). To counteract mycotoxicosis in farm animals, the European Commission (EC) regulation on additives for use in animal nutrition was amended and a new regulation (EC No 386/ 2009) in the category of technological feed additives defines a new functional group of feed additives as “substances for reduction of the contamination of feed by mycotoxins: substances that can suppress or reduce the absorption, promote the excretion of mycotoxins or modify their mode of action.” (EC 2009). Consequently, several research groups have examined the potential of mineral adsorbents to bind mycotoxins *in vitro*, and their protective effects against mycotoxicosis *in vivo*. The topic has been extensively reviewed by Huwig et al. (2001), Döll and Dänicke (2004), Avantaggiato et al. (2005), Kolosova and Stroka (2011), Di Gregorio et al. (2014), Zhu et al. (2016), Wielogórska et al. (2016), Peng et al. (2018), and Vila-Donat et al. (2018); a summary of recent studies (2014–2019) carried out on the efficacy of mineral adsorbents to alleviate mycotoxicosis in broiler chicken is shown in Table 1. Data on mycotoxin adsorption by mineral adsorbents with other animal species during the time period are scarce in the scientific literature. Other studies have also shown that mineral adsorbents may not be effective and can induce deleterious effects in farm animals when supplemented with feeds (Dwyer et al. 1997; Watts et al. 2003. Döll and Dänicke 2004; Lemke et al. 2001; Khatoon et al. 2018). As these agents are naturally abundant, inexpensive, and can be chemically modified to further increase mycotoxin-adsorption capacity, leading to an extensive use in livestock feeding, this review aims to highlight adverse effects in farm animals caused by the supplementation of animal feeds with mineral adsorbents and consequences of their interaction with veterinary substances and micronutrients in feeds.

**Table 1:** *In vivo* studies on the adsorption of mycotoxins in broiler chicken fed contaminated diet

Adsorbents	Level (g/kg)	Mycotoxins (mg/kg)	Duration (days)	Main effect of the inclusion of a mineral adsorbent to the contaminated feed	References
Bentonite	10	AFB_1_ (0.02) ZEN (2) OTA (0.1)	42	No significant difference was observed in the overall performance of bentonite-treated birds	Pappas et al. (2014)
Montmorillonite	5	T-2 (4) HT-2 (0.66)	42	Significantly improved growth, serum biochemical parameters and reduced the level of toxins in tissues.	Yang et al. (2014)
Bentonite	7	AFB_1_ (0.02)	21	No significant differences were seen in terms of feed intake and biochemical parameters measured	Dos Anjos et al. (2015)
Diatomaceous earth	7.5	AFB_1_ (0.02)	21	Significantly decreased feed intake, body weight and serum concentration of glucose, albumin and protein	Anjos et al. (2016)
Calcium bentonite	2	AFB_1_ (200, 400, 600, and 1800)	21	Treatment reduced the accumulation of AFB_1_ residues in the liver	Fowler et al. (2015)
Bentonite	3.7, 7.5	AFB_1_ (0.1, 0.2, 0.6) OTA (0.15, 0.3, 1)	21	Both concentrations of bentonite ameliorated toxic effects of 0.1 and 0.2 mg/kg AFB_1_, but no significant effects on OTA-treated birds	Bhatti et al. (2016)
HSCAS	5	AFB_1_ (2) FB_1_ (10)	37	HSCAS did not have any significant effect on reduced body weight and feed intake induced by mycotoxins	Sobrane Filho et al. (2016)
Bentonite	10	AFB_1_ (0.1) OTA (0.1)	42	Significantly reduced OTA concentration in liver and breast muscle by 4-fold and completely removed AFB_1_ residues.	Pappas et al. (2016)
Bentonite	5	AFB_1_ (2)	21	Improved growth performance and increased liver and kidney weight.	Shannon et al. (2017)
Bentonite	5	AFB_1_ (0.1, 0.2, 0.6); OTA (0.15, 0.3, 1)	42	Decreased 41% of AFB_1_ residues in the liver of broiler chicken	Bhatti et al. (2017)
HSCAS	3	AFB_1_ (0.04)	21	Significantly improved growth performance, digestibility and reduced AFB_1_ in liver and kidney.	Liu et al. (2018)
Bentonite	5, 10, 20	OTA (0.15, 0.3, 1)	42	No significant effect on total antibody, immunoglobulin titres and lymphoproliferative responses.	Khatoon et al. (2018)
Aluminosilicate	1	AFB_1_ (2, 4)	21	Increased body weight and feed efficiency as well as haematological parameters and serum proteins.	Nazarizadeh and Pourreza (2019)
Modified HSCAS	5	T-2 (6)	14	Prevented T-2 toxin-induced decreased body weight, feed intake, protein and total calcium and phosphorus	Wei et al. (2019)

### Composition and structure of clay minerals

Clays are fine graded (average particle size < 0.002 mm) natural rock or soil material that exhibit plasticity when moistened or non-plastic and hard when dried (Andrade et al. 2011). Aluminosilicates are the largest and most important class of clay minerals, composed of silica, alumina, and significant amounts of alkaline and alkaline earth ions (Moreno-Maroto and Alonso-Azcárate 2018). Within this class, phyllosilicates and tectosilicates have received much scientific attention because they have a wide range of applications (Srinivasan 2011; Ghadiri et al. 2015).

The basic structure of phyllosilicates is based on tetrahedral sheets (T) composed of individual tetrahedrons which share three out of four oxygens, and octahedral sheets (O) composed of individual octahedrons that share apical oxygen and hydroxyl anion groups with cations such as aluminum and magnesium. The stacking of both sheets determines the chemistry and crystallography of each phyllosilicate. The T-O ratio of layer structure is used to classify phyllosilicates into 1:1 (T-O) and 2:1 (T-O-T) (Wang et al. 2015). Example of phyllosilicates include montmorillonite, illites, bentonite, and kaolinite. Tectosilicate have a three-dimensional framework structure wherein all the four oxygens of tetrahedron are shared with other tetrahedra (Alaniz et al. 2012); thus, the T-O ratio is 1:2. Examples of tectosilicates include zeolite, quarts, and feldspar. A summary of main clay minerals or mineral adsorbents currently used for sequestering mycotoxins is shown in Table 2.

**Table 2:** Summary of physicochemical properties of mineral adsorbents commonly used for adsorbing mycotoxins (Deepthy and Balakrishnan 2005; Lantenois et al. 2008; Pushcharovsky et al. 2016)

Adsorbent	Structure	CEC (cmol/kg)	Surface area (m^2^/g)	Mode of formation
Bentonite	2:1 Lattice	53–83	370–490	Alteration of volcanic ash in marine environment or silica bearing rocks such as granite and basalt.
Kaolinite	1:1 Lattice	3–15	5–20	Rock weathering or by hydrothermal process at high temperature or at low temperature by the alteration of primary minerals (such as feldspar).
Montmorillonite	2:1 Lattice	80–100	70–800	Weathering products in soils at moderately high temperature (200 °C)
Palygorskite	2:1 Lattice	4–40	300–600	Alteration of precursor minerals or by precipitation from rock solution.
Activated carbon	Pore	–	300–4000	Pyrolysis of different kinds of organic materials such as lignin, coconut shell, peat, hard and soft wood, lignite coal and carbonaceous materials.
Zeolite (Clinoptilolite)	1:2 Lattice	180–600	500–700	Rock interaction with aqueous solution or fluid in a wide variety of geochemical environments.

Although phyllosilicates and tectosilicates are composed of O and T sheets as predominant building blocks, the physicochemical properties of these minerals including charge, polarity, expandability, cation exchange capacity (CEC), pH, particle size, surface area, swelling ability, and adsorption capacity are dependent on structure, composition, and geographical origin (Deepthy and Balakrishnan 2005; Ito and Wagai 2017). In terms of natural abundance, a large percentage of mineral adsorbents are found mostly in twenty-three countries: Mexico, Germany, Armenia, Turkey, Italy, Uzbekistan, Azerbaijan, Kazakhstan, Ukraine, Turkmenistan, Bulgaria, USA, Czech Republic, South Africa, Moldova, Greece, Indonesia, Japan, Australia, Kyrgyzstan, Russia, Belarus, Tajikistan (EHC 2005).

### Potential adverse effects of mineral adsorbents

Mineral adsorbents are added to animal feed as a non-nutritive additive, to prevent lump formation (anti-caking agent and coagulant), improve farm animal performance and bind or reduce mycotoxins (Kolosova and Stroka 2012). The mechanism of action for sequestering mycotoxins remains controversial, and six mechanisms have been proposed: selective chemisorption, electron donating, hydrogen bonding, furan ring bonding, ion interactions and coordination between exchange cations and the carbonyl groups. However, most researchers believe the process is physical adsorption by ion exchange reaction and electrostatic interaction (Thimm et al. 2001; Deng et al. 2010; Wang et al. 2018). Similar mechanisms have also been proposed for the adsorption of nutritional content in animal diets including proteins (Ralla et al. 2010; Alam and Deng 2017), micronutrients (Schmidhalter et al. 1994; Barrientos-Velázquez et al. 2016), as well as veterinary drugs (Devreese et al. 2013). Furthermore, rate of adsorption is dependent on the origin and physicochemical properties of adsorbents and takes place predominantly in the acidic pH range (Deng et al. 2010).

Currently, bentonite, kaolinite, clinoptilolite, palygorskite, and montmorillonite are commercially available in natural and modified forms. Modified adsorbents tend to have higher mycotoxin-sequestering capacity, due to alteration in charge and surface properties of natural adsorbents, using modifiers such as acids, alkalis, and organic cations—ACO, VTMS, HDTM, BAC, OBAC, and CMAB (Baglieri et al. 2013; Nones et al. 2016; Wang et al. 2018). Concerning the safety of these agents, published studies are either contradictory or lack some degree of accuracy in their assessments. Negative effects induced in farm animals due to interaction with veterinary substances and micronutrients in feed as well as *in vitro* and *in vivo* toxicity of natural and modified mineral adsorbents are discussed as follows.

### Micronutrients

Micronutrients including iron, iodine, calcium, magnesium, selenium, zinc, and vitamins are essential minerals or elements required in minute quantities (less than 100 mg/kg per day) by animals for proper functioning of enzymes and hormones, to maintain growth and development (Smith et al. 2018). Clays are considered to be a source of minerals for animals due to the possession of an anionic framework with well-defined microstructures containing chemical elements mostly alkali metal ions and trace elements (Bhaskaran and Gupta 2006; Suzanne et al. 2017). Moreover, these minerals are exchangeable during ion-exchange process that is largely influenced by pH and temperature, leading to either bioavailability of minerals or deficiency in the gastrointestinal tract (ingesta) of animal fed feed supplemented with mineral adsorbents (Lukman et al. 2013). For instance, the hydrochloric acid in the stomach (low pH) and bile salts in the intestine (high pH) may change the physiochemical properties of mineral adsorbents thereby enhancing their ion-exchange capacity. This process may lead to the release of minerals from the surface of the adsorbents into the surrounding milieu, thus increasing the concentration of minerals (in addition to mineral content of feed) in the systemic circulation and subsequent accumulation in the body (Park et al. 2002; Mascolo et al. 2004; MambaI et al. 2010). Also, the ion-exchange process, particularly for adsorbents with a high CEC, may lead to adsorption of minerals and nutrients from feed, resulting in the deficiencies of micronutrients such as iron, potassium, and vitamins in farm animals (Ralla et al. 2010).

Several publications have reported symptoms of vitamin A deficiency in chickens given feed supplemented with 0.5–3% bentonite (Briggs and Spivey 1999; Laughland and Phillips 2000; Hashemipour et al. 2010). Moreover, Erwin et al. (1998) demonstrated through an *in vitro* study that sodium bentonite has a strong affinity for pure carotene and can as well bind non-carotenoid pigments in feed (Erwin et al. 1998). With regard to trace elements, adverse effects observed in farm animals have been attributed to the imbalance between dietary concentration of trace elements in feed and the amount of trace elements in the mineral adsorbents (Thilsing et al. 2007; Grosicki and Rachubik 2010; Yang et al. 2017).

Supplementation of feed with either 10 or 20 g/kg of palygorskite significantly decreased lead and copper accumulation in breast and thigh muscles of broiler chickens (Cheng et al. 2016). Correspondingly, inclusion of 0.5–2% zeolite and bentonite to chicken diet decreased serum levels of zinc, copper, and manganese, while aluminium concentration was significantly increased (Chung et al. 1990; Ivan et al. 1992; Utlu et al. 2007; Schwaller et al. 2016; Toprak et al. 2016). European Food Safety Authority Panel on Additives and Products used in Animal Feed (EFSA FEEDAP Panel) warned of a potential binding of manganese when bentonite is used at a dosage higher than 0.5% in feeds (EFSA FEEDAP Panel 2011a). Hooda et al. (2004) and Seim et al. (2013) also demonstrated different mechanisms by which mineral adsorbents (bentonite and halloysite) can inhibit the absorption of dietary iron *in vitro* (Hooda et al. 2004; Seim et al. 2013).

As the levels at which minerals occur in clays varies and dependent on geographical origin, it is essential to know the appropriate amount of clay and trace elements to be included in feed, to ensure animal welfare and productivity is not impaired by dietary mineral imbalances. Furthermore, a quality control system that includes adequate milling, cleaning, drying, and sieving as well as analysis of elemental composition, purity, and microbiological examination of final product must be established.

### Veterinary substances

Due to non-specific effects of mycotoxin binders, EFSA has recommended evaluation of oral veterinary drugs in feed supplemented with a mycotoxin binder, to prove its safety towards binding of medical substances (EFSA FEEDAP Panel 2011a, b). Interaction of mineral adsorbents with veterinary substances has been reported for antibiotics such as tilmicosin, tylosin, paromomycin, doxycycline, and coccidiostats such as monensin and salinomycin. Goossens et al. (2012) studied the effects of bentonite on the oral bioavailability of doxycycline in pigs fed trichothecene-contaminated feed. The authors observed an increased plasma concentration of doxycycline administered as a single bolus in animals fed 100 μg/kg of T-2 and 1 mg/kg DON-contaminated diet when compared to control group. They suggested a complex interaction leading to increased oral bioavailability of antibiotics may occur when animals are given mycotoxin-contaminated diet, mycotoxin binders, and antibiotics concurrently (Goossens et al. 2012). Efficacies of monensin and salinomycin to prevent coccidiosis in chickens were reduced in the presence of 0.5% sodium bentonite (Gray et al. 1998). Addition of bentonites to chicken diet has been shown to be incompatible with the use of robenidine and is expected to reduce the effectiveness of other coccidiostats at levels higher than 0.5% (EFSA FEEDAP Panel 2011a, b). A bacteriostatic feed additive, tylosin, was unable to prevent airsacculitis and other clinical symptoms caused by *Mycoplasma gallisepticum* in broiler chickens fed a diet supplemented with 2% bentonite (Shryock et al. 1994).

An *in vitro* model simulating intestinal barrier was designed by Devreese et al. (2013) using porcine small intestinal epithelial cell line-J2 (IPEC-J2), to study efficacy and drug interaction testing of mycotoxin binders. The model was used to examine the passage of tylosin through the intestine in the presence of 1% bentonite. Bentonite interacted with tylosin and decreased its passage through IPEC-J2 (Devreese et al. 2013). Also, an *in vitro* study on adsorption of doxycycline by six different adsorbents (four montmorillonite based-clay, sepiolite and leonardite-based binder) showed that less than 25% of the initial concentration of doxycycline was detected after 4 h of incubation at 37 °C, suggesting that 75% of doxycycline was adsorbed by mineral adsorbents (De Mil et al. 2015). Furthermore, the* in vitro* results were validated using two of the montmorillonites-based clays, to study the pharmacokinetics and oral bioavailability of doxycycline *in vivo*. Results showed that the two binders significantly lowered the area under the plasma concentration–time curve of doxycycline by less than 60% when compared with the control group. Similar *in vivo *result was reported by Osselaere et al. (2012); they observed a significant alteration in pharmacokinetic profiles and oral bioavailability of oxytetracycline and amoxicillin. Additionally, significant concentration of oxytetracycline was found in the kidneys of treated birds (Osselaere et al. 2012) compared to control group. Taken together, if a mycotoxin binder decreases or enhances the oral absorption of drugs, it may have a significant consequence on animal health, withdrawal time of the antibiotics and potentially public health in terms of exposure to antibiotic residues.

### *In vitro *toxicity

The *in vitro* toxicity of mineral adsorbents has been widely studied. Due to the potential of mineral adsorbents to enter the body through different routes including inhalation, ingestion, and dermal penetration, cell lines such as keratinocytes, alveolar macrophages, erythrocytes, endothelial, hepatocytes, epithelial, and fibroblasts have been used to investigate toxic effects of mineral adsorbents (Elmore 2003; Maisanaba et al. 2015a). These cell lines represent major organs where adsorbent particles are localized and accumulated when humans and animals are exposed to clay particles via different routes Michel et al. 2014; Connolly et al. 2019; Boim et al. 2019). Several *in vitro* toxicity studies of clay minerals have suggested an interaction or crosstalk between the surface of clay particles and cellular receptors (Verma et al. 2012; Michel et al. 2014). Furthermore, techniques such as fluorescence microscopy, transmission electron microscopy, time-lapse video microscopy, and histocytological staining have been utilized to show uptake of clay particles by macrophages and lymphocytes (Bowman et al. 2011; Kuhn et al. 2014; Connolly et al. 2019) as well as internalization by cell types such as keratinocytes and hepatocytes, through endocytosis and micropinocytosis pathways (Michel et al. 2014; Kuhn et al. 2014; Castro-Smirnov et al. 2017; Connolly et al. 2019). Also, accessory minerals such as quarts and metal oxides including TiO_2_ and ZnO contained within clays have been suggested to be responsible for toxicity induced in different cell lines, with degree of toxicity dependent on size, shape, surface properties, and chemical composition of the adsorbent (Geh et al. 2006; Li et al. 2010).

To elucidate mechanism of toxicity, different biomarkers such as lactate dehydrogenase leakage, reactive oxygen species generation, superoxide dismutase inhibition, and malondialdehyde release have been assayed (Li et al. 2010; Zhang et al. 2010; Baek et al. 2012; Lordan et al. 2010; Maisanaba et al. 2014). Furthermore, comet, Ames, and CBMN assays were used to detect DNA damage and chromosomal loss (Li et al. 2010; Maisanaba et al. 2015a). Mineral adsorbents used for the assays are generally prepared by making up a suspension and measuring the absorbance to determine the concentration, followed by a multi-step ultrasonication at 40% vibration amplitude to disperse the suspension. Finally, the known stock solution is serially diluted in cell culture medium and vortexed vigorously before being applied to cells. The culture medium without mineral adsorbent is used as the control. The majority of publications on the topic found mineral adsorbents to be toxic while a smaller number observed little or no effects.

An investigation of short and long term toxicity of montmorillonite in human normal intestinal cells (INT-407) by Baek et al. (2012) revealed that montmorillonite (20–1000 μg/mL) can inhibit cell proliferation, induce oxidative stress and membrane damage between 24 and 72 h, with more remarkable cytotoxicity after long-term exposure (10 days) (Baek et al. 2012). Apoptosis and oxidative stress were also observed in human B lymphoblast cells exposed to 120 μg/mL and 240 μg/mL of natural bentonite and bentonite modified with 10–15% of H_2_SO_4_, within 24 h of exposure (Zhang et al. 2010). Furthermore, oxidative stress induced by modified bentonite was significantly higher than that of natural bentonite (Zhang et al. 2010). In terms of cell viability, CHO (ovary) and HepG2 (liver) cells exposed to montmorillonite (100–1000 μg/mL) exhibited a reduced viability and cytotoxic effects in a dose-dependent manner after 12–24 h of exposure (Li et al. 2010; Nones et al. 2015). Houtman et al. (2014) and Maisanaba et al. (2017) also observed reduced cell viability in Caco-2 (intestine) and HepG2 cell lines exposed to adsorbents modified with HDTM, ACO, and VTMS at 24 and 48 h of exposure (Houtman et al. 2014; Maisanaba et al. 2017). Other researchers (Verma et al. 2012; Maisanaba et al. 2015b) did not observe any reduction in viability of Caco-2, human umbilical vein endothelial (HUVEC), and A549 (human alveolar epithelial) cells exposed to mineral adsorbents. This may be due to the concentrations tested and type of adsorbents used. For instance, Maisanaba et al. (2015b) used a very low concentration range (0–8 μg/mL) of montmorillonite and both Verma et al. (2012) and Liu et al. (2012) used halloysite clays (aluminosilicates) (Verma et al. 2012; Maisanaba et al. 2015b).

Regarding the genotoxicity of mineral adsorbents, EFSA FEEDAP Panel found algae interspaced bentonite to be non-genotoxic or mutagenic at the highest concentration tested, 250 μg/mL (EFSA FEEDAP Panel 2016). In a recently published opinion (EFSA FEEDAP Panel 2017), it was also reiterated that bentonite is non-genotoxic. Likewise, Geh et al. (2006), Li et al. (2010), and Maisanaba et al. (2014) did not observe any genotoxic effects when cells were exposed to natural or unmodified mineral adsorbents. Nevertheless, contrary results were observed in the case of modified adsorbents. Exposure of HepG2 cells to 15.6 μg/mL organo-modified montmorillonite significantly increased the frequency of micronuclei by 2.7-fold compared to control group using both comet and CBMN assays. Additionally, genes involved in DNA damage, metabolism, and oxidative stress were upregulated (Maisanaba et al. 2016). Similarly, HepG2 and Caco-2 cells exposed to 88 μg/mL and 141 μg/mL respectively of modified montmorillonite showed a significant increase in DNA damage after 48 h of exposure (Sharma et al. 2010; Maisanaba et al. 2013).

*In vitro* toxicity testing plays an important role in the analysis of toxic effects of chemical substances and helps to identify potentially hazardous chemicals in the environment in a rapid and cost-effective way (Maisanaba et al. 2015b). *In vitro* toxicological studies of mineral adsorbents suggest adsorbents, especially modified ones, may induce dose-dependent deleterious effects on various cell lines. With respect to the dosage used in the studies, there were challenges in describing the *in vitro* kinetics in the culture medium and actual kinetics when cells in the target tissue are exposed to the chemical substances. As *in vitro *models do not accurately determine the consequences of *in vivo* exposure because of differences in cell phenotype, immune systems, diverse protein reactions, intracellular signaling and fluidic environment (Baek et al. 2012), it is imperative to also evaluate toxicity of mineral adsorbents in animal models, taking into consideration their various applications in livestock nutrition.

### *In vivo *toxicity

As stated earlier, several studies have shown that mineral adsorbents can alleviate negative effects induced by mycotoxins in farm animals (Table 1), other studies however, have reported undesirable effects in animal groups fed diets (with or without mycotoxins) supplemented with mineral adsorbents. Khatoon et al. (2018) observed a significant reduction in the total immunoglobulin, lymphoproliferative response and total antibody in birds fed OTA-contaminated diet (0.15–1.0 mg OTA/kg) and 0.5–2% of bentonite clay (Khatoon et al. 2018). Significant decrease in creatinine, uric acid, and cholesterol serum levels were also seen in broiler chickens fed AFB_1_ plus 0.25% and 0.5% clinoptilolite (Maciel et al. 2007). Three milligrams per kilogram of AFB_1_ plus sodic montmorillonite (0.25 and 0.5%) significantly protected against the toxic effects of AFB_1_ in pigs; however, 0.5% of sodic montmorillonite significantly reduced the level of serum phosphorus (Franciscato et al. 2006).

In layer birds, inclusion of 2% clinoptilolite to diet contaminated with 2.5 mg AFB_1_/kg significantly decreased egg weight and egg yolk index (Rizzi et al. 2003). Significant decrease in triiodothyronine and thyroxine hormones were also observed in birds fed 2.5–5% of bentonite and 1 mg/kg of AFB_1_ (Eraslan et al. 2005). Addition of 4 g/kg of aluminosilicates to female weaned piglets diet contaminated with 8.6 mg DON/kg and 1.2 mg ZEN/kg significantly decreased feed intake and serum concentration of cholesterol. Furthermore, treated animals exhibited significant increase in the activities of aspartate transaminase and γ-glutamyltransferase as well as serum concentration of albumin (Döll et al. 2005). The efficacy of low-pH montmorillonites modified with HDTMA and HDA to sequester ZEN was investigated *in vivo *using uterine weight of mice (Lemke et al. 2001). Supplementation of 0.5% modified montmorillonites with ZEN-contaminated feed did not only reduce the body weight of animals but also enhanced the toxicity of ZEN (increase in uterine weight). The authors concluded that alkylamine groups may promote the uptake of ZEN from a contaminated diet and result in an enhanced toxicity (Lemke et al. 2001). In all the studies outlined, negative effects of adsorbents were observed not only in animal groups fed mycotoxin-contaminated diet with mineral adsorbents but also in group fed mycotoxin-free diet plus mineral adsorbents.

Many of the studies that reported protective actions of mineral adsorbents against toxic effects of mycotoxins *in vivo* focused mainly on zootechnical parameters such as feed intake and body weight gain without investigating the potential negative (unwanted) effects of mineral binders, as previously discussed by Döll and Dänicke (2004) (Döll and Dänicke 2004). The studies generally used three experimental groups: a negative control group (mycotoxin-free diet), positive control group (mycotoxin-contaminated diet), and treated group (mycotoxin-contaminated diet and mineral adsorbent), to demonstrate the efficacy of the mineral binders. However, a group of animals were not included (mycotoxin-free diet with mineral adsorbent) to investigate nonspecific effects of mineral binders, which may occur independently of mycotoxin contamination. Therefore, the safety or efficacy of such mineral binders has not been proven in a satisfactory manner. Other studies that investigated the effects of dietary mineral adsorbents (not as mycotoxin binder) have observed various negative effects.

Pigs fed a diet supplemented with 2.5% and 5% montmorillonite experienced hepatic histological changes including swelling, vacuolar and vesicular degeneration. Furthermore, antioxidant capacity, glutathione peroxidase and average daily feed intake were severely affected compared to control group (Zhao et al. 2017). The authors concluded that inclusion of montmorillonite to a diet at a concentration above 1% may not be safe for starter pigs. Prvulović et al. (2008) also investigated the effects of dietary supplementation of 0.5% clinoptilolite on performance and biochemical parameters of pigs. They found a significant increase in body weight gain of pigs in the first 90 days; however, after 120 days, there was a significant decrease in growth rate as well as increased activity of aspartate aminotransferase in serum of clinoptilolite-treated group (Prvulović et al. 2008). In commercial layers, Berto et al. (2013) found that 0.5% inclusion of clinoptilolite to feed led to a decrease in animal performance and eggshell quality (Berto et al. 2013). Similarly, the addition of 1–3% of sodium bentonite decreased specific gravity, yolk color index, feed consumption, and egg production compared to birds fed control diet (Roland 1988; Hashemipour et al. 2010).

The global occurrence of mycotoxins in feed ingredients is of great concern as it is considered to be a major risk factor for animal performance and human health. One of the strategies for mitigating the occurrence of mycotoxins in feed is the inclusion of mineral adsorbents to feed. Several authors have proven these substances to be effective in alleviating mycotoxicosis in farm animals fed contaminated feeds. The FEEDAP Panel have also assessed the efficacy and safety of natural bentonite, they concluded that bentonite with following composition: ≥ 70% smectite (dioctahedral montmorillonite), < 10% opal and feldspar, and < 4% quartz and calcite is effective for binding AFB_1_ and safe for all animal species when used at a maximum level of 20,000 mg/kg in complete compound feed. Several of these products have been approved by EFSA and are available on the European market as either feed additives or digestibility enhancers. However, in other countries, there is no regulation on the use of these products, and they are listed as raw material catalogue such as bentonite, kaolinite, HSCAS, and palygorskite. Moreover, very limited information is available on their composition or physicochemical properties. Therefore, utilization of such adsorbents as feed additives must be carefully considered by farmers and animal nutrition companies.

With regard to modified mineral adsorbents, very few researchers have investigated the safety of these products and both their efficacy and safety have not been assessed by the FEEDAP Panel. As it is essential to verify the potential of a mineral adsorbent to adsorb mycotoxins in vitro and in vivo, their safety and potential interaction with nutrients and veterinary substances in feeds must also be considered, using a complete experimental design: non-contaminated diet (negative control); mycotoxin-contaminated diet (positive control); non-contaminated diet with the mycotoxin binder and mycotoxin-contaminated diet with mycotoxin binder.

In summary, available data have shown that both natural and modified adsorbents or mycotoxin binders can induce cytotoxic effects including oxidative stress, reduction in cell viability, apoptosis, and DNA damage. They can bind essential micronutrients and vitamins in feed leading to reduced feed conversion, immunosuppression, and low productivity in livestock animals. Moreover, they can interact with veterinary drugs, which may cause a decline or an increase in the oral absorption of drugs, leading to a potential therapy failure and higher levels of antibiotic residues in foods of animal origin. Mineral adsorbents may also contain variable amounts of accessory minerals (quarts, nontronite, erionite), heavy metals (lead, copper, cadmium), dioxins, and trace elements, which can induce toxicity in livestock animals as well as alter serum mineral profile and activities of enzymes such as glutamate dehydrogenase, aminotransferase, creatinine, and glutathione peroxidase.

## Abbreviations

VTMS: Vinyltrimethoxysilane
HDTM: Hexadecyltrimethyl
BAC: Benzalkonium chloride
CMAB: Cetyltrimethylammonium bromide
OBAC: Octadecyldimethyl benzyl ammonium chloride
ACO: Acetylcholine chloride
HDA: Hexadecylamine
CHO: Chinese hamster ovary
HDTMA: Hexadecyltrimethylammonium
CBMN: Cytokinesis block micronucleus cytome
HSCAS: Hydrated sodium calcium aluminosilicates
